# Social inequalities in early childhood language development during the COVID-19 pandemic: a descriptive study with data from three consecutive school entry surveys in Germany

**DOI:** 10.1186/s12939-023-02079-y

**Published:** 2024-01-04

**Authors:** Stephanie Hoffmann, Mira Tschorn, Jacob Spallek

**Affiliations:** 1 Department of Public Health, Brandenburg University of Technology Cottbus-Senftenberg, Senftenberg, Germany; 2 Lausitz Center for Digital Public Health, Institute of Health, Brandenburg University of Technology Cottbus-Senftenberg, Senftenberg, Germany; 3 Social and Preventive Medicine, Department of Sports and Health Sciences, Intra-Faculty Unit Cognitive Sciences, Faculty of Human Science, University of Potsdam, Potsdam, Germany

**Keywords:** Health inequalities, Child development, COVID-19 pandemic, School entry survey

## Abstract

**Background:**

Social health inequalities are still of great public health importance in modern societies. The COVID-19 pandemic may have affected social inequalities in people's health due to containment measures. As these measures particularly affected children, they might have been particularly vulnerable to increased social inequalities. The aim of the study was to describe health inequalities during the pandemic based on language delay (LD) in children in order to inform public health interventions for a population at risk of long-term health and education inequalities.

**Methods:**

Data of 5–7 year old children from three consecutive school entry surveys in the German federal state of Brandenburg were used, including data compulsorily collected before the pandemic (2018/2019: *n* = 19,299), at the beginning of the pandemic (2019/2020: *n* = 19,916) and during the pandemic (2020/2021: *n* = 19,698). Bivariate and multivariate binary regression analyses [OR, 95% CI] cross-sectionally examined the relationship between the prevalence of LD [yes/no] and social inequalities, operationalized by family socioeconomic position [SEP low/middle/high], migration background [native-German language/non-native German language] and length of kindergarten attendance [< 4 years/ ≥ 4 years]. Factors contributing to inequality in LD were examined by socioeconomic stratification.

**Results:**

Cross-sectionally, LD prevalence has decreased overall (2018/2019: 21.1%, 2019/2020: 19.2%, 2020/2021: 18.8%), and among children from both high SEP and native German-speaking families. As LD prevalence increased among children from families with low SEP and remained stable among non-native German speakers, social inequalities in LD prevalence increased slightly during the pandemic i) by low SEP (2018/2019: OR = 4.41, 3.93–4.94; 2020/2021: OR = 5.12, 4.54–5.77) and ii) by non-German native language (2018/2019: OR = 2.22, 1.86–2.66; 2020/2021: OR = 2.54, 2.19–2.95). During the pandemic, both migration background and kindergarten attendance determined LD prevalence in the high and middle SEP strata. However, the measured factors did not contribute to LD prevalence in children from families with low SEP.

**Conclusion:**

Social inequalities in LD increased due to opposing trends in prevalence comparing low and high SEP families. To promote health equity across the life course, early childhood should be of interest for tailored public health actions (e.g. through targeted interventions for kindergarten groups). Further analytical studies should investigate determinants (e.g., parental investment).

## Introduction

Since the beginning of 2020, the Coronavirus 2019 disease (COVID-19) has spread worldwide, changing young children’s social lives and their health-related living conditions [1]. During the pandemic, public health measures were implemented to mitigate the spread of the ‘severe respiratory coronavirus 2’, the agent of COVID-19. Until late 2020, no licensed vaccine was available [2] and, as part of non-pharmaceutical containment measures in Germany, schools and kindergartens were closed to varying degrees and social activities of children were reduced in amount and type (e.g., stay-at-home restrictions, social distancing rules) [3, 4].

Children’s development can be defined by „the extent to which individual children […] are able or enabled to […] develop the capacities that allow them to interact successfully with their biological, physical, and social environments” [5]. Children aged 0 to 6 years (i.e., early childhood [6]) may be particularly vulnerable to inadequate development of language due to altered social interactions caused by public health interventions during the pandemic, as this stage of life is both sensitive to environmental exposures [7] and critical for language development [8]. For example, Singh et al. [9] suggest that wearing face masks may impair the ability of two-years-old children to recover spoken language. Further, educational disruption during the pandemic (e.g., due to kindergarten closures) may be associated with poor language development of three-years-old children [10]. Poor language development in early childhood might lead to language delay compared to peers in phonology (sounds), lexicon (vocabulary), and syntax (grammar) [11–13]. Language delay in early childhood is recognized as a public health problem, as previous empirical evidence suggested both immediate and long-term effects of individuals development, health, and educational trajectories [14, 15]. For example, Boyle [16] summarized longitudinal studies of language disorders in preschool children that suggest lasting impact on “literacy, behavior, social development into adulthood”. Beitchman et al. [17] suggested poorer self-rated physical health among adults with childhood language disorders.

To date, the literature has proposed a socioecological theoretical framework for studying the determinants of developmental delays in children in the context of epidemiological research (such as Bronfenbrenner's ecological model (1996)), which allows for the consideration of "large-scale sociohistorical events" such as the COVID-19 pandemic [3, 18]. In short, Bronfenbrenner's ecological model describe determinants of health in terms of ecological contexts, each of which entails resources and burdens on health, and all of which could be affected by a socio-historical context [19]. Bronfenbrenners model describes different ‘levels’ of ecological contexts in which the individual is embedded. In early childhood, these include the family and the kindergarten. Previous studies on early childhood health support socioecological approaches to studying inequalities in children's health [20, 21]. In case of the COVID-19 pandemic, studies suggest that children's language development in early childhood may be affected by individual level factors [11] as well as by both family level disruption and educational level interruption [3]. At an individual level, previous studies have found that boys' gender is associated with language delay [15]. Empirical evidence from before the pandemic shows that the extent of children's vocabulary depends on the socioeconomic position of their families [22]. Hoff et al. [23] found that socioeconomically disadvantaged children have a higher burden of language delays than children from better-off families even before the pandemic. The authors argued that low parental education [22] and socioeconomic differences in parental language [24] contribute to socioeconomic inequalitites in children's language development. 

Pandemic-related changes in the labour market in Germany include less or no work at all [25], with workers with a low level of education were more often affected than their better-off colleagues were [26]. In addition, parents had to reorganise everyday family life (e.g. raising children [27]) during the pandemic, partly due to the closure of kindergartens. Thus, the pandemic may have exacerbated already existing social inequalities [28], which may affect children's language development.

**Fig. 1:**
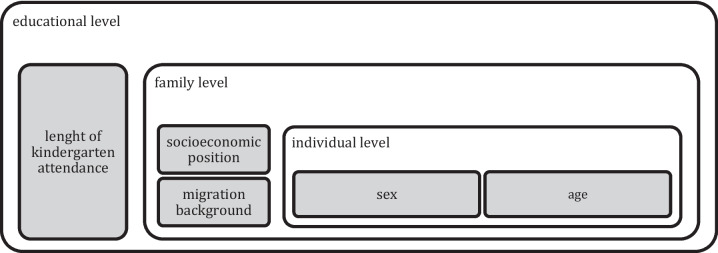
Theoretical framework for this study on social inequalities in the prevalence of language delay during the COVID-19 pandemic, based on Bronfenbrenner's ecological model (1996)

Since the social living situation could buffer or exacerbate the pandemic’s challenges to child language development [3], the current literature is insufficient to inform public health interventions on the development of social inequalities in the prevalence of early childhood language delays during the pandemic for the following reasons. First, recent prevalence estimates of language delay have relied on selective samples [10, 29]. Second, few empirical studies of child development in Germany have been conducted that aimed to describe developmental outcomes during the pandemic rather than social inequalities [30]. However, Weyers and Rigó [31], for example, empirically investigated social inequalities in children's language problems, the results of which indicate a worsening during the pandemic, regardless of the social living situation of the neighbourhood deprivation that “served as a proxy of the family SEP” [31]. Following the recommendations for descriptive epidemiology by Fox et al. [32], the present study considered ecosocial determinants [33] (see Fig. 1) and described social inequalities in health based on children’s language delay during the COVID-19 pandemic. The study used three non-selective, non-clinical samples of children from the German federal state of Brandenburg, addressing the following research questions (RQ):

1. Did social inequalities in the prevalence of language delay persist, increase or decrease during the pandemic?
2. Which risk or protective factors contribute to the prevalence of language delay in social groups at the beginning of and during the pandemic?

## Methods

### Secondary data of School Entry Examinations (SEE)

We used population-based data from school entry examinations conducted in the German federal state of Brandenburg in three consecutive birth cohorts: before the pandemic (survey I: 2018/2019; planned school start: 2019), at the beginning of the pandemic (survey II: 2019/2020; planned school start: 2020), and during the pandemic (survey III: 2020/2021; planned school start: 2021). By law, a school entry examination is required for all children living in the federal state of Brandenburg [34], resulting in complete cohort data in survey I and survey II. Due to the temporary restructuring of the Brandenburg public health services during survey III, 1.6% of eligible children could not be included in the SEE. SEE data during the pandemic were collected by public health services from winter 2020 to spring 2021. Thus, children in the third survey (2020/2021) experienced at least one year of their early childhood in the early phase of the pandemic in Germany, when vaccines were not yet available and non-pharmaceutical comprehensive containment measures were subject to law regulations including kindergarten and workplace closure [35]. SEE data refer mainly to preschool age, which has been identified as a particularly “sensitive phase of language development” [36, 37]. However, due to the study design and availability of data, it was not possible to study children living under containment measures during earlier stages of language development. Data was collected with parent questionnaires (paper–pencil) and validated child development tests. The data were provided by the ‘State Office for Occupational Safety, Consumer Protection and Health of the federal State of Brandenburg' (LAVG) in compliance with national data protection regulations. Ethical approval was not obtained as completely anonymized secondary data were used.

### Variables

#### Outcome: delayed language development

The children's language development [delayed yes/no] have been assessed by the German public health services with validated instruments of the ‘Social Paediatric Screening of Developmental Status for School Entry’ (SOPESS) [38]. In order to assess language delay using SOPESS, four tests are usually administered during the SEE: Test 1 on plural formation, Test 2 on preposition formation, Test 3 on the use of so-called pseudowords and Test 4 on phonological processing. All answers in the tests are scored. A language delay is documented if less than 50% of the points are scored correctly in i) Test 1 or ii) Test 2 or in iii) Test 3 with Test 4 [39].

Delayed language development is assessed taking into account the child's native language. This information is used to operationalise the migration background [38]. If German is not the native language (e.g., child with a migration background), public health services qualitatively and routinely assess German language skills based on spontaneous speech. Children who speak German with considerable difficulty and mistakes do not have to take test 1 and test 2. The SOPESS is considered the gold standard for school entry examinations in Germany [40]. The investigated domain of language has been proven (reliability (Cronbach's alpha) = 0.55—0.81 [41]; convergent validity e.g. with SETK 3–5 for measuring the language development of three- to five-year-old children [42]: lower sensitivity = 0.177–0.762, higher specificity = 0.651 and 0.899) [38].

The framework for this study was based on Bronfenbrenner's ecological model, so we considered variables at different ‘levels’ of ecological contexts [43].

#### Individual level (covariates)

Individual level variables were children’s sex [male/female] and children’s age [≤ 6 years, 6–7 years, ≥ 7 years].

#### Family level

Children´s social inequalities were operationalized on the family level.

#### Family socioeconomic position

The family socioeconomic positions (SEP) were based on parent-reported data on maternal and paternal primary education [1 no graduation/ < 10th grade; 2 10th grade, 3 > 10th grade] and their employment status [1 not employed, 2 employed]. In case of single parent household, the values are considered twice. To form a composite SEP variable, which is also routinely used in public social reporting on socioeconomic inequalities in health in the German federal state of Brandenburg, a sum score was formed, ranging from 4 to 10 and then divided into three categories [9–10 points: high SEP; 7–8 points: middle SEP; 4–6 points: low SEP] [44].

#### Migration background

The child's migration background was determined on the basis of parents’ native language. This information was obtained from the parent questionnaire with the item “Which native language does your child speak?” [German/ other than German/ child grows up bilingual] (Ministry of Labor, Social Affairs, Health, Women and Family of the federal state of Brandenburg, 2020).

##### Educational level (length of kindergarten attendance)

The children’s social inequalities were operationalized at the educational level, based on the information provided by the parents in survey II and survey III to the question "Since when has your child been attending a kindergarten?" [since more than 4 years/since less than 4 years]. For day care centres in Germany, this indicates whether or not the child attended a special part of the day care centre for children under the age of three (so-called ‘Kinderkrippe’) [45]. This information was not available for survey I, as the operationalisation was different in surveys II and III.

### Statistical analyses

All analyses were conducted using R (4.2.2). For the cross-sectional analyses, children without missing data in any of the variables were selected as the analysis sample. We followed a two-step approach:

- Step 1 (RQ1): Confidence intervals (95%) for the prevalence (in percent) of language delay were calculated separately for each survey year. To test bivariate associations with language delay, chi-square tests were conducted with factors at the family level (SEP, migration background) and at the educational level (length of kindergarten attendance). Odds ratios and their confidence intervals (95%) were calculated on the basis of two-by-two tables in order to present effect sizes. Logistic regression analyses were conducted for each survey year to examine multivariate associations between language delay and both family level and educational level factors. The point prevalence of each survey was presented using line graphs for a) overall participants and b) according to the social factors if significant differences have been found.
- Step 2 (RQ2): We repeated the logistic regression models stratified by SEP as the factor that proved most relevant to the prevalence of language delay (Step 1). The variables that were found to be significantly related to language delay were used to test stratified risk or protective factors.

Testing assumptions for the logistic regression for each survey: We found no evidence of significant multicollinearity with variance inflation factors (VIF) below 1.05 for all predictors (age, gender, SEP, migration background, length of kindergarten attendance). In addition, the average VIF for all predictors was < 1.01 and the tolerance statistics were > 0.98 [46, 47].

*P*-values were considered significant at *p* < 0.05.

## Results

Table 1 shows characteristics of the children who participated in the school entry examinations, (1) before the pandemic (survey I 2018/2019: *n* = 23,997), (2) at the beginning of the pandemic (survey II 2019/2020: *n* = 24,850) and (3) during the pandemic (survey III 2020/2021: *n* = 24,788). Due to missing data on socioeconomic position, migration background (i.e., non-German native language) and/or length of kindergarten attendance, the statistical analyses considered *n* = 19,299 (2018/2019), *n* = 19,916 (2019/2020) and *n* = 19,698 (2020/2021) children (see Table 2).

**Table 1:** Characteristics of survey participants

		**Year of survey**					
		**2018/2019** **Pre-pandemic**		**2019/2020** **Pandemic start**		**2020/2021** **Pandemic**	
**N**		23,997		24,850		24,788	
		**N**	**%**	**N**	**%**	**N**	**%**
**Sex**	Girls	11,322	47.2	11,867	47.7	11,758	47.4
	Boys	12,675	52.8	12,983	52.3	13,030	52.6
**Age**	≤ 6.0 years	15,077	62.9	15,049	60.6	13,729	55.4
	< 7.0 years	8,689	36.2	9,550	38.4	10,753	43.4
	> 7.0 years	231	0.96	251	1.0	306	1.2
**SEP index**	High	9,853	41.1	10,802	43.5	10,966	44.3
	Middle	9,473	39.4	9,277	37.3	8,756	35.3
	Low	2,050	8.5	1,930	7.7	1,766	7.1
	Missing information	2,621	10.9	2,841	11.4	3,300	13.3
**Native language**	German	21,308	88.8	22,055	88.7	21,542	86.9
	Other (mb)	1,236	5.2	1,338	5.4	1,559	6.3
	Bilingual	884	3.7	770	3.1	896	3.6
	Missing information	569	2.4	687	2.8	791	3.2
**Kindergarten**	< 4 years	n.a	n.a	4,789	19.3	2,362	9.5
	> 4 years	n.a	n.a	17,285	69.6	19,927	80.4
	Missing information	n.a	n.a	2,776	11.2	2,499	10.1

*n.a.* not available (In 2018/2019, the ‘kindergarten’ variable was operationalised differently than in 2019/2020 and 2020/2021.); *mb* migration background, *SEP* socioeconomic position

**Table 2:** Prevalence of language delay depending on the year of the survey

Year of survey					
**2018/2019** *N* = 19,299		**2019/2020** *N* = 19,916		**2020/2021** *N* = 19,698	
Delay (%)	95% CI	Delay (%)	95% CI	Delay (%)	95% CI
21.1	0.205–0.217	19.2	0.186- 0.197	18.8	0.183–0.194

*N* drawn as a sample from all participating children, *CI* confidence interval (wald)

### Point prevalance estimates for each survey (RQ1)

While the point prevalence of language delay at school entry was 21.1% before the pandemic, it was 19.2% at the start of the pandemic and 18.8% during the pandemic in the three samples analysed (see Table 2).

#### Family socioeconomic position

Children from socioeconomically advantaged families had significantly lower prevalence rates in each of the three surveys (I: 14.3%; II: 13.7%; III: 13.0%) compared to children from both middle socioeconomic families (I: 24.2%; II: 21.4%; III: 21.7%) and socioeconomically disadvantaged families (I: 42.4%; II: 42.5%; III: 43.4%). Bivariate analyses revealed higher odds ratio of language delay in children from socioeconomically disadvantaged backgrounds compared to children from high SEP families in each survey (I: OR_lowSEP_ = 4.41, 3.93–4.94; II: OR_lowSEP_ = 4.66, 4.15–5.24, III: OR_lowSEP_ = 5.12, 4.54–5.77). The point prevalence of language delay decreased in each survey for children from high socioeconomic backgrounds, while it increased for children from low SEP.

#### Migration background

A language delay occurred more frequently in children with a migration background (i.e., non-German native speaker) (I: 36.0%; II: 35.2%; III: 35.5%) than in children with German as their native language (I: 20.2% II: 18.2; III: 17.8%). Accordingly, the bivariate analyses for each survey showed an increased probability of language delay for non-German native speaker (I: OR = 2.22, 1.86–2.66; II: OR = 2.44, 2.09–2.86; III: OR = 2.54, 2.19–2.95). The point prevalence of language delay decreased in each survey for children of German native speakers, while it remained for children with a migration background.

#### Length of kindergarten attendance

The proportion of children with delayed language development was lower among those who attended kindergarten for more than four years than among those who attended the kindergarten for less than four years (II: 26.8%_<4 years_, 17.3%_≥4 years_; III: 27.5%_<4 years_, 17.9%_≥4 years_). Bivariate analyses again showed that a decreased probability of a language delay if the kindergarten was attended for more than four years (II: OR = 0.56, 0.52–0.61; III: OR = 0.58, 0.52–0.64).

**Fig. 2:**
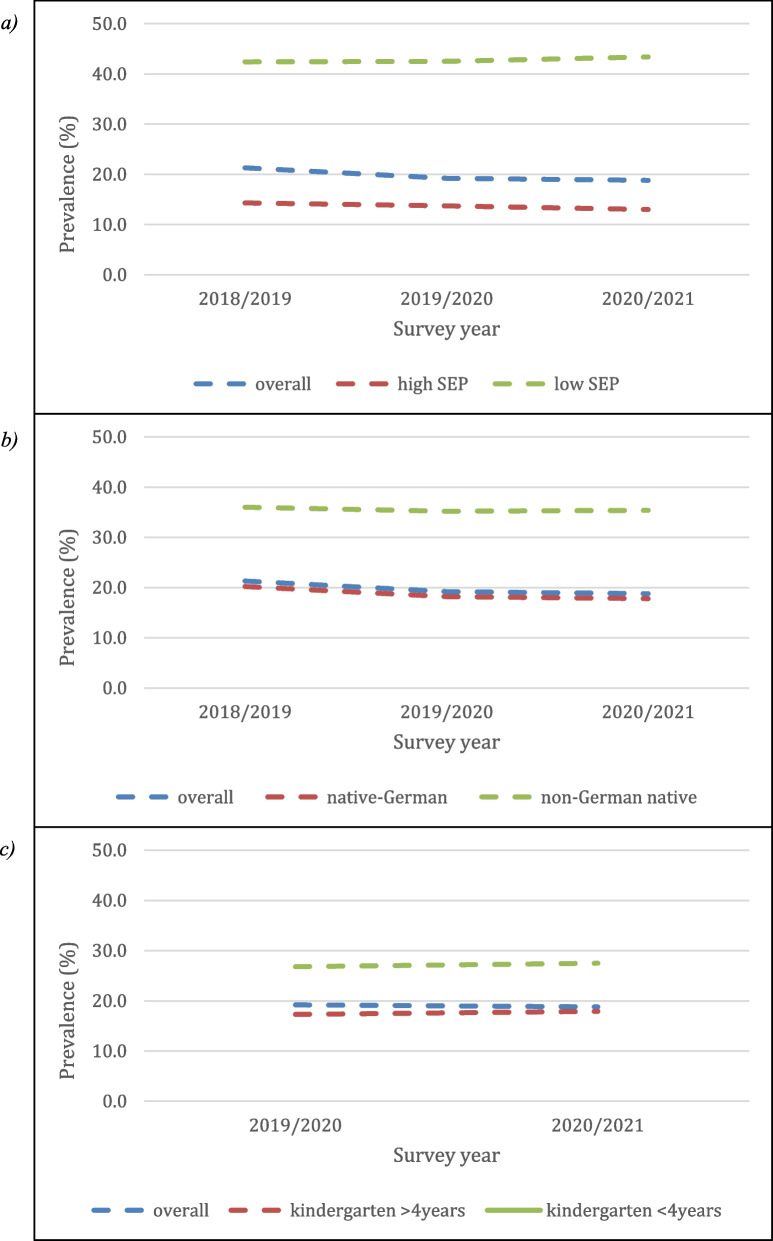
Point prevalence of language delay in each survey overall and with (**a**) socioeconomic position and with (**b**) native language (migration background) and with (**c**) length of kindergarten attendance. Note: SEP = socioeconomic position

Figure 2a, b and c display cross-sectional point prevalence rates according to family level and educational level factors.

Multivariate binary logistic regression models revealed associations with all social indicators used, which were examined separately for each survey.

Table 3 illustrates the results of the bivariate and multivariate analyses.

**Table 3:** Point prevalence of language delay depending on individual, family and educational factors

		**Survey year 2018/2019**^**a**^			**Survey year 2019/2020**^**b**^			**Survey year 2020/2021**^**c**^		
		N	% Delay	OR (95% CI)	N	% Delay	OR (95% CI)	N	% Delay	OR (95% CI)
Bivariate associations										
**Sex**	Girls^Ref^	9,112	17.4	-	9,606	15.7	-	9,433	15.3	-
	Boys	10,187	24.4	1.53***(1.43–1.64)	10,310	22.4	1.54***(1.44–1.66)	10,265	22.0	1.56***(1.46–1.68)
**Age**	< 6.0 years^Ref^	12,224	21.5	-	12,316	19.7	-	11,098	20.1	-
	< 7.0 years	6,941	19.9	0.90** (0.84–0.97)	7,453	17.7	0.87***(0.81–0.95)	8,411	16.6	0.79***(0.73–0.85)
	> 7.0 years	134	52.2	3.98***(2.80–5.71)	147	51.7	4.36***(3.12–6.14)	189	40.7	2.72***(2.00–3.69)
**Language**	German^Ref^	18,111	20.2	-	18,642	18.2	-	18,221	17.8	-
	other (mb)	569	36.0	2.22***(1.86–2.66)	753	35.2	2.44***(2.09–2.86)	825	35.5	2.54***(2.19–2.95)
	bilingual	619	34.9	2.11***(1.78–2.52)	521	31.9	2.10***(1.74–2.54)	652	25.5	1.57***(1.31–1.89)
**SEP index**	high^Ref^	9,089	14.3	-	9,948	13.7	-	10,231	13.0	-
	middle	8,558	24.2	1.91***(1.77–2.07)	8,416	21.4	1.71***(1.58–1.85)	8,019	21.7	1.84***(1.71–2.00)
	low	1,652	42.4	4.41***(3.93–4.94)	1,552	42.5	4.66***(4.15–5.24)	1,448	43.4	5.12***(4.54–5.77)
**Kindergarten**	< 4 yrs^Ref^	-		-	3,989	26.8	-	1,817	27.5	-
	> 4 yrs	-		-	15,927	17.3	0.56***(0.52–0.61)	17,881	17.9	0.58***(0.52–0.64)
Multivariate logistic regression										
	language^[other (mb)]^	-		1.80***(1.49–2.16)	-		1.83***(1.55–2.16)	-		1.97***(1.67–2.32)
	SEP index^[middle]^	-		1.97***(1.82–2.13)	-		1.73***(1.60–1.87)	-		1.89***(1.75–2.05)
	SEP index^[low]^	-		4.23***(3.76–4.76)	-		4.04***(3.58–4.55)	-		4.71***(4.16–5.33)
	kindergarten^[≥4 yrs]^	-		-	-		0.75***(0.68–0.81)	-		0.85**(0.75–0.96)
		-		R2 Tjur	-		R2 Tjur	-		R2 Tjur
				0.056			0.058			0.060

Multivariate analyses adjusted for age, sex and bilingual language. *mb* migration background, *N* sample size, *Ref* reference, *OR* odds ratio, *CI* confidence interval, *SEP* socioeconomic position, *yrs* years; * *p* < .05, ** *p* < .01, *** *p* < .001

^a^ 2018/2019 considered children: *n* = 19,299

^b^ 2019/2020 considered children: *n* = 19,916

^c^ 2020/2021 considered children: *n* = 19,698

### Risk or preventive factors contributing to language delay at the beginning and during the pandemic (RQ2)

Step 1 revealed that low socioeconomic position was the most important factor for language delay, with prevalence rates of 42.5% (survey II) and 43.4% (survey III). By socioeconomic stratification, migration background and length of kindergarten attendance were examined as social risk or preventive factors for survey II (at the beginning of the pandemic) and survey III (during the pandemic). In survey I, the length of kindergarten attendance was measured differently.

#### Migration background

At the beginning of the pandemic, migration background (operationalised here by ‘other than German native language’) was linked to a decreased probability of language delay in socioeconomically disadvantaged children (OR_lowSEP_ = 0.66, 0.47–0.91) and with an increased likelihood among both other socioeconomic strata (OR_highSEP_ = 2.90, 2.26–3.69; OR_middleSEP_ = 2.43, 1.81–3.23). Multivariate analyses of data during the pandemic found these associations for families in high and middle SEP, but not for children from lower SEP (OR_lowSEP_ = 1.13, 0.83–1.55).

#### Length of kindergarten attendance

Kindergarten attendance of more than four years was associated with lower likelihood of language delay in all socioeconomic strata at the beginning of the pandemic (OR_highSEP_ = 0.70, 0.60–0.81; OR_middleSEP_ = 0.79, 0.69–0.90; OR_lowSEP_ = 0.75, 0.61–0.93). Multivariate regression analyses also found these associations for families with high and middle SEP during the pandemic, but not for socioeconomically disadvantaged children (OR_lowSEP_ = 1.03, 0.81–1.32).

Table 4 contains the odds ratios of the logistic regressions stratified by SEP.

**Table 4:** Mutlivariate regression analyses based on socioeconomic stratification

		**Socioeconomic position index**					
		**High**		**Middle**		**Low**	
2019/2020^a^		N	%Delay	N	%Delay	N	%Delay
		9,948	13.7	8,416	21.4	1,552	42.5
Multivariate logistic regression		OR (95% CI)		OR (95% CI)		OR (95% CI)	
	Language^[other (mb)]^	2.90***(2.26–3.69)		2.43***(1.81–3.23)		0.66* (0.47–0.91)	
	Kindergarten^[≥4 yrs]^	0.70***(0.60–0.81)		0.79***(0.69–0.90)		0.75**(0.61–0.93)	
		R2 Tjur		R2 Tjur		R2 Tjur	
		0.026		0.021		0.030	
2020/2021^b^		N	%Delay	N	%Delay	N	%Delay
		10,231	13.0	8,019	21.7	1,448	43.4
Multivariate logistic regression		OR (95% CI)		OR (95% CI)		OR (95% CI)	
	Language^[other (mb)]^	2.52***(1.97–3.20)		2.27***(1.71–3.00)		1.13 (0.83–1.55)	
	Kindergarten^[≥4 yrs]^	0.78* (0.63–0.96)		0.81* (0.68–0.98)		1.03 (0.81–1.32)	
		R2 Tjur		R2 Tjur		R2 Tjur	
		0.022		0.019		0.013	

Analyses adjusted for age, sex and bilingual language; As the variable 'kindergarten' was operationalised differently in 2018/2019 (survey I) than in 2019/2020 and 2020/2021, survey I was not considered valid for the stratified analyses

*mb* migration background, *N* sample size, *Ref* reference, *OR* odds ratio, *CI* confidence interval, *yrs* years * *p* < .05, ** *p* < .01, *** *p* < .001

^a^ 2019/2020 considered children: *n* = 19,916

^b^ 2020/2021 considered children: *n* = 19,698

## Discussion

We examined social inequalities in child development, examined by the prevalence of language delays, based on data from three consecutive school entry surveys in the German federal state of Brandenburg before the pandemic (survey I), at the beginning of the pandemic (survey II) and during the pandemic (survey III). The prevalence of language delay decreased slightly over time overall and for children from high and middle SEP families, while opposite trends were observed for children from low SEP families. Among children with German native language, the prevalence of language delay decreased slightly, while it remained stable among children with a migration background. For the length of kindergarten attendance, we found similar associations with language delay in all surveys.

### Increasing social inequalities in the prevalence of language delay during the pandemic

With the present operationalization, our results point to an increasing social inequality in language delay due to family level factors (SEP, migration background).

First, the point prevalence of delayed language development has decreased in the three cross-sectional samples. This result is in part unexpected, as one previous German study found an increase in prevalence rates of language delays among preschool children during the pandemic [30]. In particular, Bantel et al. [30] found an increasing proportions of children with language support needs during the pandemic in the German city of Hannover, which was also analyzed with data from school entry examinations. However, these results are only comparable to a limited extent, as their data include a large number of children with a migration background (43%; here: < 10% in all surveys) and/or with low household education (29%; here family SEP < 10% in all surveys), which may imply systematic bias. In addition, stratification was by migration background rather than SEP, and the prevalence of language problems (i.e., different outcomes) was higher among children with a migration background [30].

Second, the multivariate regression models adjusted for individual level factors (age, sex), confirmed cross-sectional associations with the two family level factors measured (SEP, native language) in all surveys. The prevalence rates in the pre-pandemic survey and in the pandemic survey decreased in children with high SEP, while they increased in children with low SEP. Likewise, the odds ratio of delayed language among children from socioeconomically disadvantaged backgrounds compared to children with high SEP families was marginally lower before the pandemic than during the pandemic. Further, the proportion of children with a language delay in each sample decreased in children with German as a native language, while it remained stable for children with a migration background Accordingly, the odds ratios differed slightly with regard to each survey. Our findings are consistent with previous literature on family SEP and migration background, both of which have been associated with delayed language development in preschool children [23, 48–51].

Third, multivariate regression models adjusted for individual level (age, sex) and family level factors (SEP, native language) confirmed cross-sectional associations with length of kindergarten attendance for survey II and survey III. Children who attended kindergarten for more than four years were less likely to have a language delay than children who attended kindergarten for fewer years. However, the odds ratio in the surveys did not differ. Our findings are in line with previous literature suggesting association between the length of kindergarten attendance and language impairment [52].

Social inequalities in children's language delay, as measured by family SEP and migration background, increased in the pandemic survey compared to pre-pandemic data. However, this cannot be clearly attributed to pandemic measures for the following reason. The prevalence increase of language delay to the disadvantage of children from families with low SEP and children with non-German native language was already visible in survey II, while a pandemic effect is unlikely in this period (autumn 2019 to spring 2020), as Germany did not implement containment measures until March 2020 [53]. Although the association of family level factors on language delay differed only slightly between the first and the third survey, the increase of social inequalities, therefore, may be independent of the containment measures and may only have continued in the pandemic years.

### Risk and preventive factors for language delay at the beginning and during the pandemic

As family level factor, migration background (non-German native language) has found to be associated with language delays across all socioeconomic strata at the beginning of the pandemic. With varying effect sizes and directions, findings suggest that native language could be a risk factor among socioeconomically better-off families (all OR > 1) and a preventive factor among children from disadvantaged families (OR < 1). During the pandemic, migration background remained a significant risk factor for language delay in families with high and middle SEP.

As an educational level factor, length of kindergarten attendance remained a significant factor in children's language delay at the beginning of the pandemic in all socioeconomic strata and during the pandemic in high and middle socioeconomic status families, with kindergarten attendance of more than four years being a protective factor for language delay (all OR < 1).

The measured factors (native language, length of kindergarten attendance) were not associated with language delay among socioeconomically disadvantaged children in the pandemic survey. This could be a statistical artefact (e.g. an overadjustment), so an association may not be apparent [54]. However, according to the bioecological model of development [43], there may be other unmeasured individual (e.g., pre-existing health problems) [55] and social factors (e.g. family, neighbourhood) [56] that might contribute to language delay during the pandemic and require further investigation.

Since the data were collected in the German federal state of Brandenburg, the results on social inequalities in the healthy development of children must be interpreted with regard to the federal state’s social situation. In terms of the year 2018, the material prosperity of the state was considered average in Germany, with a disposable income per inhabitant of about 21,000 euros (range nationwide: 19,800–25,500 euros) and an adult unemployment rate of 6.3% (range nationwide: 2.9%-9.8%) [57]. As of 31 December 2018, 25.5% of the German population had a migration background,^1^ of which 47.6% were foreign nationals [58]. The proportion of persons with a migration background in Brandenburg was one of the lowest nationwide (< 11%), but with one of the highest proportion of foreign nationals (50- < 56%) [59]. In the survey years, 94% (2018/2019), 95% (2019/2020) and 96% (2020/2021) of three- to five-year-old children were cared for in daycare centres, whose care rates were among the highest nationwide [57].

### Strength and limitations

The use of SEE data allows for the “timely and efficient” [31] consideration of social living situation indicators to describe developmental inequalities over the course of the pandemic. Our study used data from the German federal state of Brandenburg collected in three consecutive years with a harmonized variable operationalization. During the pandemic year, almost all children were screened, resulting in unbiased SEE data with similar participant characteristics compared to previous surveys. This is a strength compared to other studies using SEE data, which may refer to a reduced number of children during the pandemic year, many of whom were children with health or social problems [30]. Another strength is that we were able to use family level SEP indicators to operationalize social disadvantage, which was in part not possible in previous studies [30, 31].

Our study has several limitations. First, we could only use the available data on family and educational level factors, which we selected based on Bronfenbrenner's ecological model [43]. Other family-related variables (i.e., income) might have produced different results [60–62]. For example, data do not include information on whether or not children grow up in single parent households, which could also be associated with both child development outcomes [31] and socioeconomic disadvantages in terms of income in Germany [63]. In addition, according to Bronfenbrenner [43], the determinants of child development may be related to the children's living environment outside the family and the kindergarten. As previous studies have found an association between developmental delays and regional socioeconomic deprivation in addition to family socioeconomic position [56] or with regard to rural–urban differences [52], further research could consider possible spatial determinants of developmental delay during the COVID-19 pandemic. Second, the available SEE variables include a SEP index whose operationalisation may not adequately represent the social situation, leading to i) unbalanced data (e.g., a kind of oversampling) and ii) biased effect estimates (e.g., collider stratification bias) [64]. In this study, a large number of migrant families in survey II were classified as having low SEP (26%; among native-German speaker 6.9%). Hence, potential moderation effects that have not been investigated here should be analysed in further studies.

Third, our analyses are descriptive in nature (i.e., explorative) with cross-sectional samples and do not allow us to draw conclusions about temporal trends during the pandemic, as SEE data collected in 2021/2022 or 2022/2023 (endemic or post-pandemic) are not yet available. Future research could, therefore, consider time-series (i.e., using pooled cross-sectional data or longitudinal data) analyses that include children with later school entry to analyse the temporal impact of the pandemic on preschool children's delayed language development. In addition, the analyses conducted here should be repeated in future years to confirm social inequalities, explore other potential determinants, and examine children who spent a greater proportion of their early language development years under pandemic influences.

Fourth, our results only apply to the social situation of the study region, which is characterised by an average material endowment, a lower proportion of people with a migration background and higher rates of childcare for 3–5-year-old children. As a result, the conclusions drawn from samples in other regions could be different.

## Conclusion

Our results show that the prevalence of language delay in children from Brandenburg, Germany, has decreased for three consecutive years. However, children were differentially vulnerable to delayed language development, as the prevalence in each year i) increased in children with low SEP and ii) persisted with migrant background, while it decreased otherwise. Migration background was a risk factor for language delay, while longer attendance of a kindergarten was protective at all time points. Socioeconomic disadvantages predominantly determined language development before and during the pandemic. During large-scale socio-historical events such as the COVID-19 pandemic, all children are of particular interest in order to prevent developmental delays and promote health and health equity throughout the life course, but children with socioeconomic disadvantage and from migrant backgrounds should be particularly targeted.

## Data Availability

All original data are on record and accessible to inspection, but are not available publicly based on local and national data protection regulations.

## Abbreviations

mb: Migration background
SEP: Socioeconomic position

## Footnotes

1 Germany's statistical definition: "A person has a migration background if he or at least one parent was not born with German citizenship”.
